# Correction: Urine peptidome in combination with transcriptomics analysis highlights MMP7, MMP14 and PCSK5 for further investigation in chronic kidney disease

**DOI:** 10.1371/journal.pone.0305767

**Published:** 2024-06-13

**Authors:** The PLOS ONE Staff

## Notice of Republication

This article was republished on June 5, 2024, to correct for column and line duplication in Table 3 introduced during the typesetting process. Please download this article again to view the correct version.

## Supporting information

S1 File Originally published, uncorrected article. (PDF)

S2 File Republished, corrected article. (PDF)
