# Supplementary material for: Correction: Urine peptidome in combination with transcriptomics analysis highlights MMP7, MMP14 and PCSK5 for further investigation in chronic kidney disease
Source: PLoS One. 2024 Jun 13;19(6):e0305767. doi: 10.1371/journal.pone.0305767 (PMC11175472; doi:10.1371/journal.pone.0305767)
Supplement: S2 File — (PDF) [file pone.0305767.s002.pdf]

PLOS ONE

RESEARCH ARTICLE

# Urine peptidome in combination with transcriptomics analysis highlights MMP7, MMP14 and PCSK5 for further investigation in chronic kidney disease

**Eleni Petra**[1,2], **Justyna Siwy**[3], **Antonia Vlahou**[2], **Joachim Jankowski**[1,4]*

**1** Institute for Molecular Cardiovascular Research, RWTH Aachen University Hospital, Aachen, Germany, **2** Center of Systems Biology, Biomedical Research Foundation of the Academy of Athens, Athens, Greece, **3** Mosaiques Diagnostics GmbH, Hannover, Germany, **4** Experimental Vascular Pathology, Cardiovascular Research Institute Maastricht (CARIM), University of Maastricht, Maastricht, The Netherlands

* jjankowski@ukaachen.de

Check for updates

OPEN ACCESS

**Citation:** Petra E, Siwy J, Vlahou A, Jankowski J (2022) Urine peptidome in combination with transcriptomics analysis highlights MMP7, MMP14 and PCSK5 for further investigation in chronic kidney disease. PLoS ONE 17(1): e0262667. https://doi.org/10.1371/journal.pone.0262667

**Editor:** Qi Wu, Aarhus University, DENMARK

**Received:** October 11, 2021

**Accepted:** January 3, 2022

**Published:** January 19, 2022

**Copyright:** © 2022 Petra et al. This is an open access article distributed under the terms of the Creative Commons Attribution License, which permits unrestricted use, distribution, and reproduction in any medium, provided the original author and source are credited.

**Data Availability Statement:** All relevant data are within the paper and its Supporting Information files.

**Funding:** J.S.: The author(s) received no specific funding for this work. E.P., A.V. and J.J.: The project is funded by the European Union's Horizon 2020 research and innovation programme under the Marie Skłodowska-Curie grant agreement No 764474 (EU-ITN CaReSyAn). J.J.: The project is funded by the 'Deutsche Forschungsgemeinschaft' (DFG, German Research Foundation) by the Transregional Collaborative Research Centre

## Abstract

Chronic kidney disease (CKD) is characterized by the loss of kidney function. The molecular mechanisms underlying the development and progression of CKD are still not fully understood. Among others, the urinary peptidome has been extensively studied, with several urinary peptides effectively detecting disease progression. However, their link to proteolytic events has not been made yet. This study aimed to predict the proteases involved in the generation of CKD-associated urinary excreted peptides in a well-matched (for age, sex, lack of heart disease) case-control study. The urinary peptide profiles from CKD (n = 241) and controls (n = 240) were compared and statistically analyzed. The *in-silico* analysis of the involved proteases was performed using Proteasix and proteases activity was predicted based on the abundance changes of the associated peptides. Predictions were cross-correlated to transcriptomics datasets by using the Nephroseq database. Information on the respective protease inhibitors was also retrieved from the MEROPS database. Totally, 303 urinary peptides were significantly associated with CKD. Among the most frequently observed were fragments of collagen types I, II and III, uromodulin, albumin and beta-2-microglobulin. Proteasix predicted 16 proteases involved in their generation. Through investigating CKD-associated transcriptomics datasets, several proteases are highlighted including members of matrix metalloproteinases (*MMP7*, *MMP14*) and serine proteases (*PCSK5*); laying the foundation for further studies towards elucidating their role in CKD pathophysiology.

## Introduction

Chronic kidney disease (CKD) is one of the leading causes of death worldwide [1]. CKD is characterized by a reduced kidney function reflected in decreased glomerular filtration rate (GFR) < 60 mL/min/1.73m$^2$, for three months or longer [2]. The cellular and molecular mechanisms underlying the development and progression of CKD are not fully understood whereas

(German Research Foundation; TRR 219; Project-ID 322900939 [C04, S03]). "The funders had no role in study design, data collection and analysis, decision to publish, or preparation of the manuscript.

**Competing interests:** J.S.: is employed by Mosaiques Diagnostics GmbH. E.P, A.V, J.J: The authors have declared that no competing interests exist. This does not alter our adherence to PLOS ONE policies on sharing data and materials.

the therapeutic methods are limited [3]. Specifically, several mechanisms including endocrine and metabolic complications as well as cardiovascular disease have a significant effect on the development and progression of CKD [2]. Furthermore, factors like proteinuria, kidney fibrosis and hypertension are associated with an increased risk of progression of CKD through the renin-angiotensin-aldosterone system (RAAS) [4].

Altered activation of proteases, breaking peptide bonds between the amino acids and generating protein fragments, are involved in key cellular mechanisms including, immune response, transcription, cell proliferation, differentiation, signalling and extracellular matrix (ECM) remodelling [5, 6]. Given the complexity of CKD pathophysiology, proteolysis is considered to play an essential role in the glomerular basal membrane breakdown, kidney damage and fibrosis [5]. Specifically, matrix metalloproteinases (MMPs), a family of zinc-containing endopeptidases, such as *MMP2* and *MMP9* have been linked to alterations of the tubular basement membrane, leading to renal fibrosis and tubular atrophy [7–9]. Furthermore, *MMP1* and *MMP7* are associated with the inflammatory process resulting in the development of renal fibrosis [7, 10–12]. The activation or inhibition of MMPs is regulated by, among others, inhibitors of metalloproteinases (TIMPs), α2-macro-globulin, netrins, tissue factor inhibitor 2 and the reversion-inducing cysteine-rich protein with Kazal motifs (RECK) [5, 7]. Disparate evidence is available supporting changes in levels or activities of various proteases in association to CKD (reviewed in [13]), yet links to specific proteolytic events are generally lacking.

Based on the novel high-throughput technologies, the discovery and development of non-invasive CKD biomarkers is highlighted increasingly in the last decade. The urinary peptidome has been extensively studied, suggesting that several urinary peptides/biomarkers can effectively detect the progression of CKD [14–19]. Notably, a panel of 273 urinary differentially excreted peptides between CKD patients and controls, known as the "*CKD273 classifier*" [16], has been used for diagnostic and prognostic purposes in all CKD stages [15, 19–21]. In addition, this urinary proteomic classifier CKD273 has been successfully used for the stratification of CKD patients in large clinical trials [22].

Prompted by this lack of links between proteases and generated peptides in association to CKD, we aimed to predict the proteases involved in the generation of CKD-associated urinary excreted peptides in a well-matched case-control study. Through cross-correlating our predictions to CKD-associated transcriptomics datasets, several proteases are highlighted including members of MMPs (*MMP7*, *MMP14*) and serine proteases (*PCSK5*), laying the foundation for further studies towards elucidating their role in CKD pathophysiology.

## Materials and methods

### Patient data and peptide data collection

Urinary peptidomics data from CKD and non-CKD individuals were retrieved from the Human Urinary Proteome database of capillary electrophoresis mass spectrometry (CE-MS) [23]. These urine sample datasets were obtained from published studies on CKD and kidney failure and described before [19, 24, 25]. The datasets were examined for the accessibility of information on kidney failure and estimated glomerular filtration rate (eGFR). In addition, since cardiovascular disease is a major confounder, data were also screened for ejection fraction (EF) and heart failure diagnosis. All participants with EF $<$ 55% and/or heart failure diagnoses were excluded from the study. The kidney function was estimated by the eGFR assessed based on ´Chronic Kidney Disease Epidemiology Collaboration´ (CKD-EPI) and the eGFR values were single measurements. Further patient characteristics and information including sex, age, body mass index (BMI), smoking status, serum creatinine, systolic blood pressure (SBP) and diastolic blood pressure (DBP) were extracted. These accessible datasets were then

divided into two groups; CKD and non-CKD individuals where CKD was defined based on kidney function (eGFR < 60 ml/min/1.73 $m^2$) including mainly diabetic nephropathy (DN), glomerulonephritis (GN) and focal segmental glomerulosclerosis (FSGS) as the associated CKD aetiologies. The non-CKD cohort had an eGFR > 60 ml/min/1.73 $m^2$. This study was conducted following the ethics approval (163/19 Ethik-Commission of the medical faculty of the RWTH Aachen), fulfilling all the requirements of the protection of the individuals participating in medical research and accordance with the principles of the Declaration of Helsinki. All data sets received were anonymized. All experiments were performed by relevant named guidelines and regulations.

## Capillary electrophoresis mass spectrometry (CE-MS)

Processing of the urine samples for CE-MS was performed as previously reported in Mischak et al. [26]. The P/ACE MDQ capillary electrophoresis system (Beckman Coulter, USA) coupled to a micro-TOF-MS (Bruker Daltonic, Germany) was utilized to perform CE-MS analysis. RAW MS data were assessed by the MosaFinder software [23]. CE-MS data normalization was performed by 29 collagen types that were not affected by the disease [27].

## Statistical analysis

The Kolmogorov-Smirnov normality test was used to determine the distribution of the urine peptidome data. Statistical analysis of the urinary peptides was performed by the non-parametric Mann-Whitney test, followed by correction for multiple testing using the Benjamini-Hochberg (BH) method. A BH adjusted P-value <0.05 was considered statistically significant. The frequency threshold of 30% in cases or controls was applied. The Spearman's rank-order correlation was performed using Python 3.8.0. Data visualization and graphical plots were created using GraphPad Prism version 7.0 (GraphPad Software, La Jolla, California, USA) and Python 3.8.0. Data are presented as mean ± SD (standard deviation) (*$P < 0.05$).

## Bioinformatic analysis

The open-source tool "*Proteasix*" (www.proteasix.org) was used to predict proteases that were potentially responsible for the generation of the CKD-associated peptides [28]. In brief, "*Proteasix*" retrieves information on the naturally occurring protease/cleavage site associations from protease databases (MEROPS, www.ebi.ac.uk/merops; UniProt, www.uniprot.org; BRENDA, www.brenda-enzymes.org) along with the cleavage site restrictions database (ENZYME database, www.brenda-enzymes.org). A list of two types of proteases is generated; 1) the observed proteases (proteases collected from the literature) and 2) the predicted proteases (the proteolysis is calculated by the MEROPS database). In this study, to improve the reliability of the data, the analysis was focused only on the observed proteases. The predicted protease activity score was calculated as previously described [17]. Pathway enrichment analysis was performed with the "*Metascape*" (www.metascape.org) with the Reactome pathway database used as an ontology source [29]. Information about the available protease inhibitors was retrieved from the MEROPS database (www.ebi.ac.uk) [30].

## Transcriptomics data

The transcriptomic data were extracted from the Nephroseq (www.nephroseq.org, University of Michigan, Ann Arbor, MI). The 16 shortlisted proteases were uploaded to the Nephroseq database and the corresponding gene expression patterns were identified in CKD databases. Four different databases where CKD, as compared to controls, were available and used: (1)

Nakagawa CKD Kidney (Discovery Set) [31], (2) Nakagawa CKD Kidney (Validation Set) [31], (3) Ju CKD TubInt [32] and (4) Ju CKD Glom [33]. The main CKD aetiologies included in datasets were DN (Ju CKD Glom; n = 12, Ju CKD TubInt; n = 17), FSGS (Ju CKD Glom; n = 25, Ju CKD TubInt; n = 17), IgA nephropathy (Ju CKD Glom; n = 27, Ju CKD TubInt; n = 25), membranous glomerulonephropathy (Ju CKD Glom; n = 21, Ju CKD TubInt; n = 18), thin basement membrane disease (Ju CKD Glom; n = 3, Ju CKD TubInt; n = 6) and vasculitis (Ju CKD Glom; n = 23, Ju CKD TubInt; n = 21). Lupus nephritis and tumour nephrectomy were excluded from Ju CKD TubInt and Ju CKD Glom studies to better match with the peptidomics data. The threshold was set at $P < 0.05$. The transcriptomic data of the four CKD databases were compared with the predicted activity of the proteases.

# Results

## Cohort characteristics

3463 datasets of the "*Human Proteome database*" were screened for the availability of renal and heart disease as well as eGFR values. Following case-control matching (no evidence for heart disease, similar age and sex distribution), two groups were generated; non-CKD (controls, with an eGFR > 60 ml/min/1.73 $m^2$, n = 240) and CKD (with an eGFR < 60 ml/min/1.73 $m^2$, n = 241). The mean eGFR value of the CKD cohort was 41.0 ml/min/1.73 $m^2$ whereas the double value (i.e. 82.0 ml/min/1.73 $m^2$) was observed for the non-CKD cohort. The majority (75%; n = 181) of CKD patients were at stage 3 (30–45 ml/min/1.73 $m^2$) followed by patients at stage 4 (n = 38, 15–29 ml/min/1.73 $m^2$) and 5 (n = 22, less than 15 ml/min/1.73 $m^2$). No significant differences between the two groups were observed in the levels of N-terminal pro-b-type natriuretic peptide (*NT-proBNP*), SBP and DBP. Serum creatinine was significantly higher in CKD patients. Clinical characteristics of the matched non-CKD and CKD patients are presented in **Table 1**.

**Table 1. Characteristics of the matched non-CKD and CKD patients.**

| | non-CKD | CKD |
|---|---|---|
| Number | 240 | 241 |
| Sex, male, (%) | 58 | 59 |
| Age, years | 71 ± 8 | 71 ± 8 |
| BMI | 26.59 ± 4.15 | 27.84 ± 4.16 |
| Smoking Status (Yes, %) | 5.1 | 8.9 * |
| eGFR (CKD-EPI), (ml/min/1.73 $m^2$) | 82.0 ± 21.4 | 41.0 ± 15.4 * |
| CKD Stage 5, (%) | N/A | 9 |
| CKD Stage 4, (%) | N/A | 16 |
| CKD Stage 3, (%) | N/A | 75 |
| NT-proBNP (pg/mL) | 203.34 ± 220.57 | 271.24 ± 159.45 |
| Serum creatinine (μmol/L) | 75.37 ± 11.82 | 106.83 ± 17.85 * |
| Systolic BP (mmHg) | 143.59 ± 18.61 | 146.58 ± 21.89 |
| Diastolic BP (mmHg) | 79.59 ± 8.99 | 79.14 ± 9.09 |

Data are presented as mean ± SD (standard deviation) or number (%). Differences between non-CKD and CKD have been evaluated by the Mann-Whitney U test (for continuous variables) or Chi-Square test (for categorical variables) and are marked with * when $P < 0.05$. Abbreviations: CKD = chronic kidney disease; BMI = body mass index; eGFR = estimated glomerular filtration rate; CKD-EPI = chronic kidney disease epidemiology collaboration; NT-proBNP = N-terminal pro b-type natriuretic peptide; BP = blood pressure.

https://doi.org/10.1371/journal.pone.0262667.t001

## Urine peptidomic analysis

In the present study, 3184 discriminatory sequenced urinary peptides were initially identified between CKD and the compared non-CKD groups. Overall, 303 urinary peptides were still significantly differentially excreted after applying multiple testing corrections (Benjamini-Hochberg (BH), P < 0.05) and the frequency threshold of 30% in cases or controls (S1 Table). A volcano plot showing the fold change difference in the 303 differentially excreted peptides is presented in Fig 1A. The majority of the differentially excreted peptides (n = 199, 65,7%) showed decreased abundance, whereas 104 (34.3%) peptides were detected with increased abundance in CKD patients versus controls. Among the former, showing the most decreased levels in CKD, were fragments of collagen types I, II, III and XI, clusterin (*CLU*), uromodulin (*UMOD*) and mucin-12 (*MUC12*); whereas peptides originated from albumin, beta-2-microglobulin (*B2M*) and alpha-1-antitrypsin (*A1AT*) were the most increased. The top 20 most up-regulated or down-regulated peptides between the two groups are presented in the heatmap (Fig 1B).

On a protein level, these 303 CKD-associated peptides originated from 69 unique protein precursors. The top 15 most frequently detected protein precursors, that covered 76%

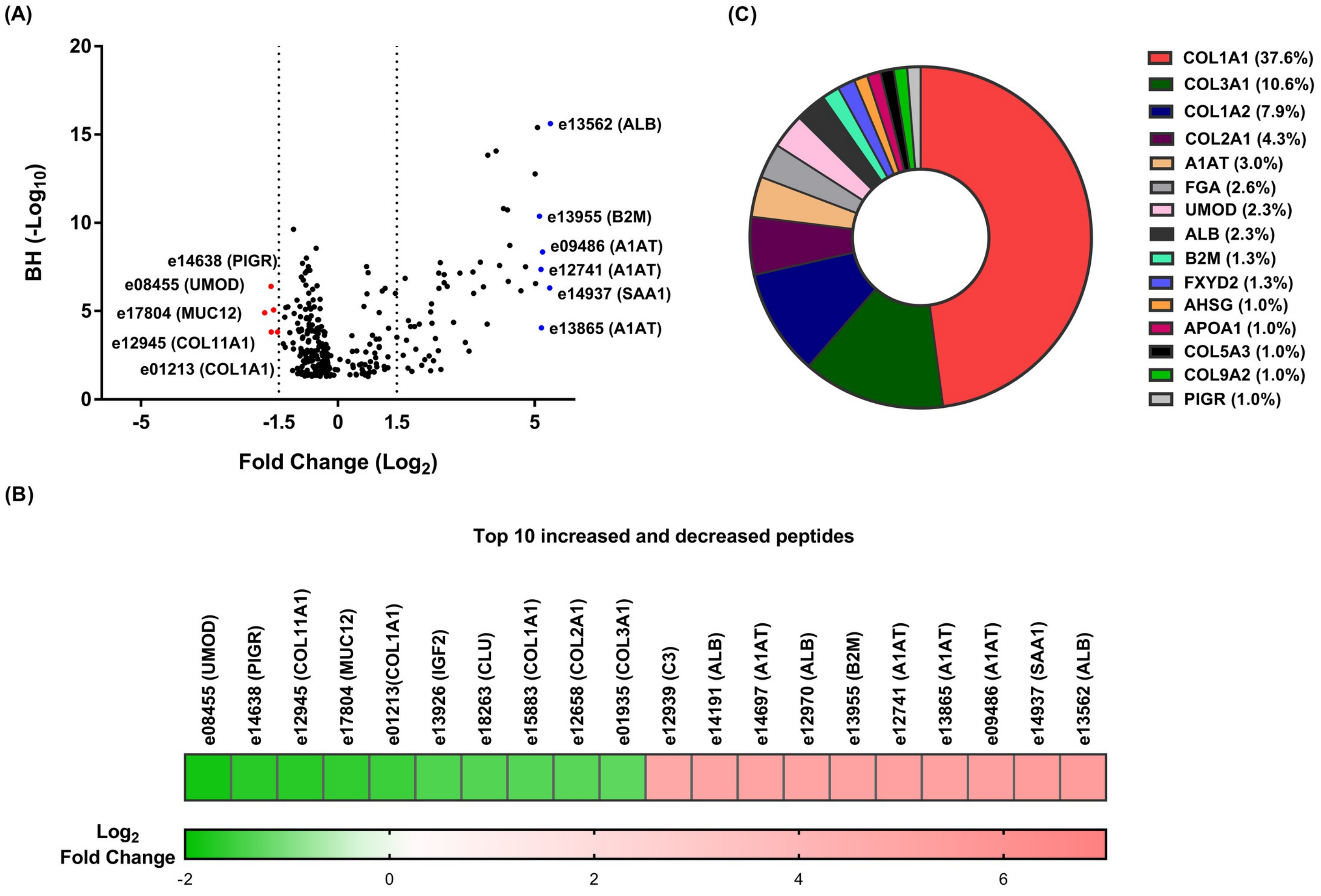


**Fig 1. Urinary peptides in CKD.** (**A**) Volcano plot showing the fold change ($\log_2$ CKD/non-CKD) plotted against the BH p-value ($-\log_{10}$) of the 303 differentially excreted CKD-associated peptides. The top five most increased and decreased abundance peptides in CKD patients are highlighted in blue (for increased) and red (for decreased) colours. (**B**) Heatmap of the $\log_2$ transformed fold change of the top 10 most increased and decreased peptides in CKD patients. In each case, the peptide codes are provided in the S1 Table. (**C**) Pie chart of the top 15 most frequently observed protein precursors, that covered 76% (n = 230) of differentially excreted CKD-associated peptides. The percentage of the differentially excreted peptides per protein precursor is presented.

https://doi.org/10.1371/journal.pone.0262667.g001

(n = 230) of differentially excreted CKD-associated peptides are presented in Fig 1C. About 31% (n = 94) of these 303 CKD-associated peptides were non-collagen and 69% (n = 209) were collagen peptides. Overall, these 209 peptides originated from 22 different collagen types with the vast majority (n = 183) deriving from fibril-forming collagens (i.e. types I, II and III).

We further investigated which of the protein precursors were represented by peptides that showed a consistent directional change in abundance. Five protein precursors including, albumin (*ALB*, number of peptides = 7), *B2M* (number of peptides = 4), apolipoprotein A-I (*APOA1*, number of peptides = 3), alpha-2-HS-glycoprotein (*AHSG*, number of peptides = 3) and alpha-1B-glycoprotein (*A1BG*, number of peptides = 2) were represented by increased peptides whereas seven protein precursors including *UMOD* (number of peptides = 8), sodium/potassium-transporting ATPase subunit gamma (*FXYD2*, number of peptides = 4), *COL9A2* (number of peptides = 3), *COL5A3* (number of peptides = 3), *COL16A1* (number of peptides = 2) and *CLU* (number of peptides = 2) were represented by peptides all at decreased abundance in CKD when compared to controls. To further investigate the association of these peptides with kidney function, we calculated the correlation between the abundance of these peptides and eGFR. A significant negative correlation was observed between all peptides originating from *ALB* (rho (range): -0.18 to -0.53, $P < 0.05$), *A1BG* (rho (range): -0.31 to -0.34, $P < 0.05$), *AHSG* (rho (range): -0.18 to -0.47, $P < 0.05$), *APOA1* (rho (range): -0.38 to -0.52, $P < 0.05$), *B2M* (rho (range): -0.17 to -0.59, $P < 0.05$) and eGFR in CKD (S2 Table), whereas the same peptides were not correlated with eGFR in the controls ($P > 0.05$). Along the same lines, a significant positive correlation was detected between all peptides of *COL16A1* (rho (range): 0.13 to 0.26, $P < 0.05$), *FXYD2* (rho (range): 0.15 to 0.33, $P < 0.05$) and eGFR in CKD patients (S2 Table).

## *In-silico* protease and protease inhibitor analysis

*In-silico* analysis by Proteasix revealed the 24 predicted proteases which possibly were involved in the endogenous cleavage of the CKD-associated peptides (Fig 2A). Of these 24, 16 proteases had a percentage of cleavage events above 1% and are presented in Fig 2A. These 16 shortlisted proteases were classified into two groups; a) MMPs including 11 family members (*MMP9*, *MMP13*, *MMP2*, *MMP14*, *MMP3*, *MMP12*, *MMP8*, *MMP20*, *MMP1*, *MMP7*, *MMP26*) and b) serine proteinases including 4 protein convertases (*PCSK6*, *PCSK7*, *PCSK4*, *PCSK5*) and kallikrein 4 (*KLK4*). About 48% of the cleavage events were predicted to be performed by *MMP9*, *MMP13 and MMP2*. However, interestingly, the proteolytic products of the 4 protein convertases and *KLK4* were among the most abundant endogenous peptides, as illustrated in the heatmap (Fig 2B). We further calculated the predicted proteolytic activity of the shortlisted proteases (**Methods and** S3 Table). Among the predicted proteases, four protein convertases, *KLK4* and *MMP7* exhibited an increased proteolytic activity whereas several MMPs (*MMP13*, *MMP9*, *MMP2*, *MMP14*, *MMP3*, *MMP12*, *MMP20*, *MMP8*, *MMP1*) were predicted at decreased activity levels, as presented in the heatmap (Fig 2C).

Pathway enrichment analysis of the 16 shortlisted proteases along with the 69 unique protein precursors of the CKD-associated peptides was performed using the Reactome pathway database. This analysis highlighted that the shortlisted proteases along with the protein precursors were significantly linked with several enriched terms including the ECM-related, metabolism, platelet signalling and vesicle-mediated transport.

Of the most pronounced, together, the predicted MMPs including *MMP9*, *MMP2*, *MMP1*, *MMP3*, *MMP12*, *MMP14*, *MMP7*, *MMP13*, *MMP8*, and *MMP20* along with several collagen types (I, II, III, IV, V, VI, VII, VIII, IX, XI, XIII, XIV, XVI, XVII, XIX, XXII and XXV) were involved in collagen-related and ECM-related pathways. The ten most significant clusters are presented in Fig 3.

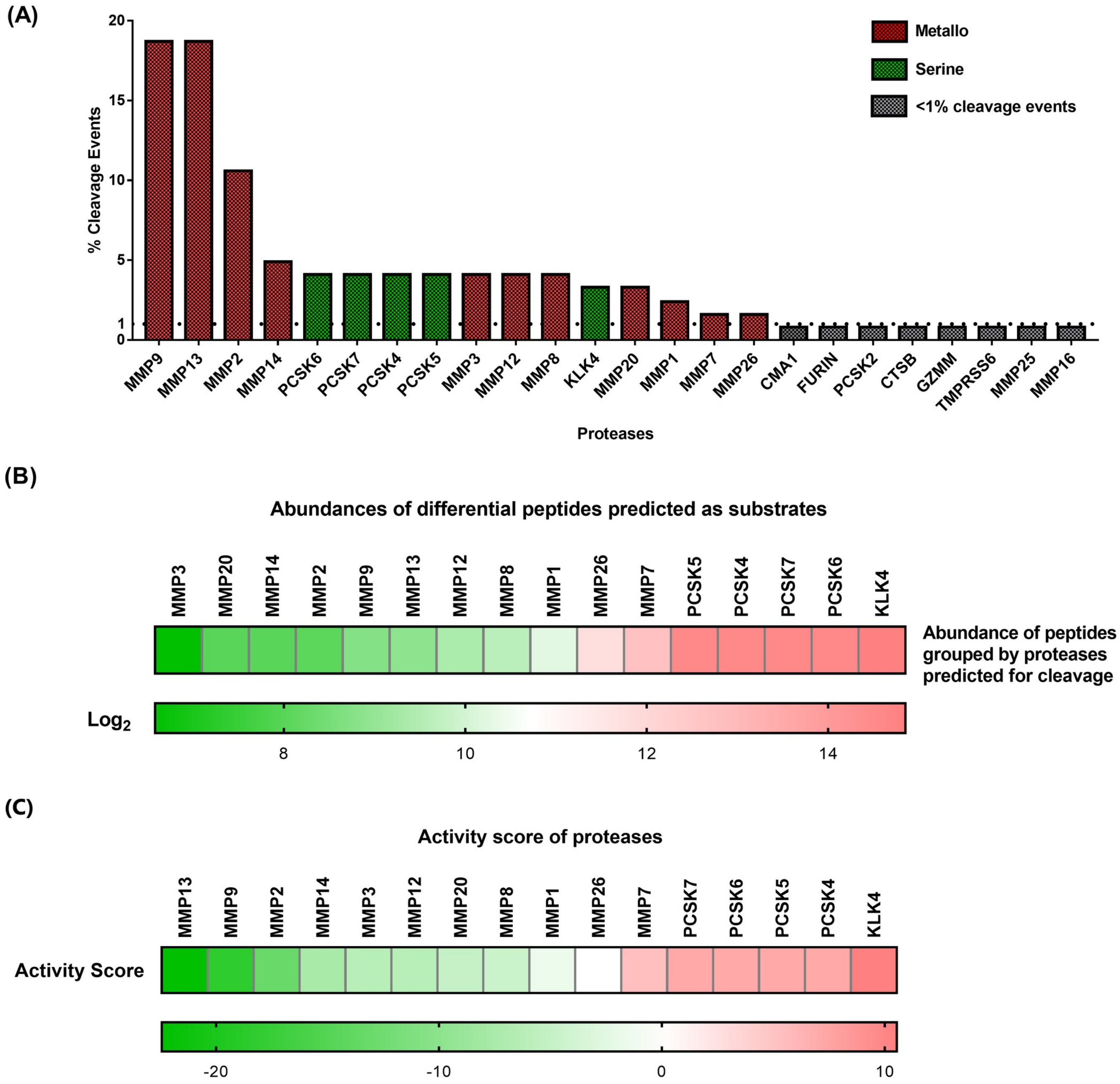


**Fig 2. *In-silico* predicted proteases.** (**A**) The 24 predicted proteases which possibly involved in the endogenous cleavage of the CKD-associated peptides are presented. Of these 24, 16 proteases had > 1% of cleavage events and are highlighted in red and green colours. Eleven metalloproteinases (highlighted in red) and 5 serine proteases (highlighted in green) are presented. (**B**) Heatmap of the $\log_2$ transformed abundance of the differentially excreted peptides grouped based on the predicted proteases. These peptides were used as substrates by the 16 shortlisted proteases. (**C**) Heatmap of the shortlisted proteases with their calculated activity score. The activated proteases are highlighted in red whereas the deactivated proteases are highlighted in green.

https://doi.org/10.1371/journal.pone.0262667.g002

Given the dependence of protease activities on protease inhibitors, we also investigated the involved inhibitors of the 16 shortlisted proteases. Along the lines of the protease analysis, information on the inhibitors was retrieved from the MEROPS protease database (www.ebi.ac.uk/merops/inhibitors/). *TIMP-1*, *TIMP-2* and *TIMP-3* were listed as regulating the activity of 8 MMPs (summarized in **Table 2**). Furthermore, four different inhibitors of serine proteases

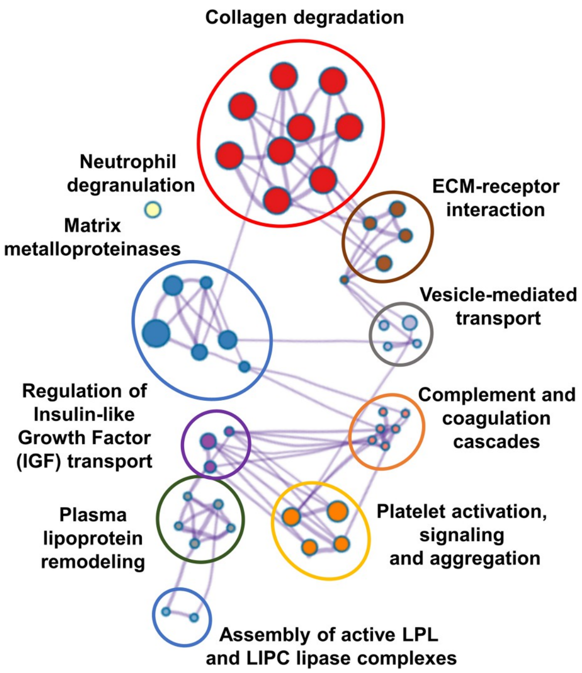


| Pathways | Associated Genes |
|---|---|
| Collagen degradation (P= 8.62E-60) | COL1A1\|COL1A2\|COL2A1\|COL3A1\|COL4A5\|COL4A6\|COL5A1\|COL5A2\|COL6A1\|COL6A2\|COL7A1\|COL8A1\|COL9A2\|COL11A1\|COL13A1\|COL16A1\|COL17A1\|COL19A1\|MMP1\|MMP2\|MMP3\|MMP7\|MMP8\|MMP9\|MMP12\|MMP13\|MMP14\|COL14A1\|MMP20\|COL5A3\|COL25A1 |
| Matrix metalloproteinases (P= 3.48E-18) | MMP1\|MMP2\|MMP3\|MMP7\|MMP8\|MMP9\|MMP12\|MMP13\|MMP14\|MMP20\|MMP26 |
| Regulation of Insulin-like Growth Factor (IGF) transport and uptake by Insulin-like Growth Factor Binding Protein (P= 5.01E-11) | AHSG\|ALB\|APOA1\|C3\|FGA\|IGF2\|MMP1\|MMP2\|SERPINA1\|SPP1\|VGF |
| Platelet activation, signaling and aggregation (P= 3.66E-10) | A1BG\|AHSG\|ALB\|APOA1\|CLU\|COL1A1\|COL1A2\|FGA\|FGB\|IGF2\|PCDH7\|SERPINA1\|TMSB4X |
| ECM-receptor interaction (P= 1.07E-09) | COL1A1\|COL1A2\|COL2A1\|COL4A5\|COL4A6\|COL6A1\|COL6A2\|COL9A2\|SPP1 |
| Plasma lipoprotein remodeling (P= 1.34E-07) | ALB\|APOA1\|APOA4\|APOC3\|PCSK6\|PCSK5 |
| Neutrophil degranulation (P= 3.19E-05) | A1BG\|AHSG\|B2M\|C3\|HSP90AB1\|KRT1\|MMP8\|MMP9\|SERPINA1\|PIGR\|S100A8 |
| Complement and coagulation cascades (P= 5.96E-04) | C3\|CLU\|FGA\|FGB\|SERPINA1 |
| Assembly of active LPL and LIPC lipase complexes (P= 3.01E-03) | APOA4\|PCSK6\|PCSK5 |
| Vesicle-mediated transport (P= 3.23E-03) | ALB\|APOA1\|COL1A1\|COL1A2\|COL3A1\|COL7A1\|MAN1A1\|SERPINA1\|SAA1\|LMAN2 |

**Fig 3. Pathway enrichment analysis.** Pathway enrichment analysis of the 16 shortlisted proteases along with the 69 protein precursors of the differentially excreted peptides was performed by the Reactome pathway database. The 10 most significant clusters are presented in different colours and the most significant term of each cluster is selected as a label. The associated proteases are highlighted in red colour.

https://doi.org/10.1371/journal.pone.0262667.g003

namely *SPINK6*, *SERPINA1*, *SERPINC1* and *SERPINF2* were linked to the activity of *KLK4* (Table 2). No data were available for the *MMP20*, *MMP26*, *PCSK6*, *PCSK7*, *PCSK4* and *PCSK5* proteases.

**Table 2. List of protease inhibitors and associated proteases (according to MEROPS).**

| Inhibitors | Proteases | | | | | | | | | |
|---|---|---|---|---|---|---|---|---|---|---|
| **Reversion-inducing cysteine-rich protein with Kazal motifs (RECK)** | MMP9 | | MMP2 | MMP14 | | | | | | |
| **Metalloproteinase inhibitor 1 (TIMP1)** | MMP9 | MMP13 | MMP2 | | MMP3 | MMP12 | MMP8 | MMP1 | MMP7 | |
| **Metalloproteinase inhibitor 2 (TIMP2)** | MMP9 | MMP13 | MMP2 | MMP14 | MMP3 | | MMP8 | MMP1 | MMP7 | |
| **Metalloproteinase inhibitor 3 (TIMP3)** | MMP9 | MMP13 | MMP2 | MMP14 | MMP3 | | MMP8 | MMP1 | MMP7 | |
| **Metalloproteinase inhibitor 4 (TIMP4)** | MMP9 | | MMP2 | MMP14 | MMP3 | | | MMP1 | MMP7 | |
| **Alpha-2-macroglobulin (A2M)** | MMP9 | | MMP2 | | | | MMP8 | MMP1 | | |
| **Pregnancy zone protein (PZP)** | MMP9 | | MMP2 | | | | | | | |
| **Testican-1 (SPOCK1)** | | | | MMP14 | | | | | | |
| **Testican-3 (SPOCK3)** | | | | MMP14 | | | | | | |
| **Serine protease inhibitor Kazal-type 6 (SPINK6)** | | | | | | | | | | KLK4 |
| **Alpha-1-antitrypsin (SERPINA1)** | | | | | | | | | | KLK4 |
| **Antithrombin-III (SERPINC1)** | | | | | | | | | | KLK4 |
| **Alpha-2-antiplasmin (SERPINF2)** | | | | | | | | | | KLK4 |

Abbreviations: MMP = Matrix metalloproteinase; KLK4 = Kallikrein-4

https://doi.org/10.1371/journal.pone.0262667.t002

## Validation of predicted proteases

To evaluate the validity of the predictions, kidney transcriptome datasets from CKD were retrieved from the Nephroseq database (www.nephroseq.org). Four datasets where the mRNA levels of expression of CKD patients were compared to healthy controls (threshold $P < 0.05$) were available in the Nephroseq database; in which the transcriptomic data related to the 16 predicted proteases were evaluated. Interestingly, the activity trend of 3 predicted proteases including *MMP7*, *PCSK5* and *MMP14* was in agreement with all transcriptomic datasets (**Table 3**). In addition, in the case of *KLK4*, the predicted activity trend was in agreement with the transcriptomic data of one dataset, whereas no data were available in the other 3 datasets. In contrast, *MMP1* and *MMP3* were found at increased mRNA levels in 3 of the transcriptomics datasets in contrary to their predicted decreased protease activity based on the peptidomics analysis (**Table 3**). In the case of the rest proteases, data were less conclusive due to inconsistencies among the different transcriptomics datasets. No transcriptomic data were available for the *PCSK4* protease (**Table 3**).

To enhance the reliability of these results, we further retrieved the relevant kidney transcriptomics data for the protease inhibitors regulating the activity of the 16 proteases from the Nephroseq database (**Table 4**). In CKD patients, *TIMP1* was found at increased mRNA levels in three of the transcriptomics datasets while *TIMP2* and *A2M* were found at increased mRNA levels in two of the transcriptomics datasets. In addition, two serpins, *SERPINC1* and *SERPINF2* exhibited decreased mRNA levels, in at least three transcriptomics datasets, in CKD versus controls (**Table 4**). We also correlated the transcriptomics data of the inhibitors with expression data as reported by scientific publications. We found that the mRNA expression trend was in agreement for 2 inhibitors, namely *TIMP1* and *SERPINA1* whereas no data were available for the other protease inhibitors in CKD patients.

## Discussion

Considering that urine is produced by the kidney, the urinary peptidome has been extensively studied to investigate kidney dysfunction. Proteases play a key role in the generation of peptides, yet their study is still limited. The presented *in-silico* analysis describes the predicted proteases involved in the proteolytic cleavage events of the CKD-associated peptides following comparison of large sample sizes of well-matched, (placing also emphasis on the main confounder -cardiovascular disease) case-control groups. The most prominent changes of the related pathways are associated with ECM remodelling, immune system and metabolism with specific peptides affected in CKD patients.

MMPs may play an important role in key cellular functions such as cell differentiation, migration, apoptosis, angiogenesis, fibrosis and inflammation through their interaction with both ECM and non-ECM substrates such as growth factors and cell adhesion molecules [7]. In the kidney, MMPs are synthesized by the tubular epithelial and the glomerular intrinsic cells [5]. *In-silico* analysis of our data pointed out that MMPs and protein convertases were possibly mainly responsible for the generation of the CKD-associated peptides. Specifically, it was predicted that for the vast majority of MMPs, except *MMP7*, a decrease in proteolytic activity is expected. These predictions were further confirmed at the mRNA level in the case of *MMP7* and *MMP14* and also enhanced by the observed increased mRNA expression levels of several inhibitors (*TIMP1*, *TIMP2*). Interestingly, some studies have reported the importance of *MMP7* in CKD, highlighting this protease as a key regulator of renal fibrosis through three main pathways, transforming growth factor-beta (TGF-β) signalling, epithelial-mesenchymal transition (EMT) and ECM deposition [34, 35]. In addition, it has been proposed that *MMP7* is activated by the Wnt/ β-catenin signalling, a pathway that is associated with kidney injury

**Table 3. List of predicted proteases with calculated activity score.**

| | Activity Score (Peptide database) | Nakagawa CKD Kidney (Discovery Set) | Nakagawa CKD Kidney (Validation Set) | Ju CKD Glom | Ju CKD TubInt | Protein expression |
|---|---|---|---|---|---|---|
| Proteases | | CKD cohort | CKD cohort | CKD cohort | CKD cohort | CKD cohort |
| MMP9 | -18.47 | Increase | Not significant* | Not significant* | Not significant* | inconclusive* |
| MMP13 | -22.44 | Increase | Increase | Not significant* | Not significant* | - |
| MMP2 | -13.28 | Decrease | Not Significant* | Increase | Increase | inconclusive* |
| **MMP14** | -7.59 | Decrease | Decrease | Decrease | Not significant* | - |
| PCSK6 | 7.08 | Decrease | Increase | Decrease | Decrease | - |
| PCSK7 | 7.08 | Decrease | Increase | Increase | Not significant* | - |
| PCSK4 | 7.08 | No data | No data | No data | No data | - |
| **PCSK5** | 7.08 | Increase | Increase | Increase | Increase | - |
| MMP3 | -6.33 | Increase | Increase | Increase | Not significant* | - |
| MMP12 | -6.33 | Decrease | Increase | Not significant* | Not significant* | Increase [61] |
| MMP8 | -4.62 | Increase | Increase | Not significant* | Not significant* | - |
| KLK4 | 10.53 | Increase | No data | No data | No data | - |
| MMP20 | -5.06 | Increase | Increase | Not significant* | Not significant* | - |
| MMP1 | -1.72 | Increase | Not Significant* | Increase | Increase | - |
| **MMP7** | 5.26 | Increase | Increase | Increase | Increase | Increase [11, 62] |
| MMP26 | 0 | Increase | Not Significant* | Decrease | Not significant* | - |

* Not significant: $P > 0.05$, inconclusive*: some studies reported increased, some decreased protein expression levels in CKD and some reported no statistically significant difference between CKD and the compared groups. Abbreviations: MMP = Matrix metalloproteinase; PCSK = Proprotein convertase subtilisin/kexin type; KLK4 = Kallikrein-4.

https://doi.org/10.1371/journal.pone.0262667.t003

[12, 36, 37]. In the case of *MMP14*, which is predicted to be at decreased activity based on the peptide and Nephroseq data, evidence that it acts as a regulatory molecule for pro-MMP2 and pro-MMP9 leading to the formation of the activated *MMP9* and *MMP2* through the complex proMMP-2/TIMP-2/MMP-14 has also been accumulated [38, 39]. Based on our results, *MMP2* and *9* may be involved in a large number of the observed peptide changes in CKD; in agreement with our predictions, it has been described that *MMP9* and *MMP2* activity is reduced with the progression of CKD due to the excessive formation of fibrosis and hypoxia [38, 40]. Overall, *MMP2* and *MMP9* may regulate the CKD pathophysiology through their interactions with tumour necrosis factors (TNFs), oxidative stress (OS), growth factors (GFs) and monocyte chemoattractant proteins (MCPs) [38]. Furthermore, it has been reported that *MMP2* and *MMP9* are differentially expressed in patients with minimal change disease, focal segmental glomerulosclerosis and membranous nephropathy [41, 42]. In addition, it has been shown that reduced expression of *MMP9* in the cytoplasm of normal tubular cells is associated with renal fibrosis, whereas *MMP2* is associated with structural changes in the tubular basement membrane leading to tubular atrophy and fibrosis [9, 41]. Regarding the inflammatory response, it has been reported that both *MMP7* and *MMP9* may increase the inflammatory process through their chemotactic effect on human dendric cells while *MMP14* may serve as

**Table 4. List of protease inhibitors with transcriptomics and protein expression.**

| | Nakagawa CKD Kidney (Discovery Set) | Nakagawa CKD Kidney (Validation Set) | Ju CKD Glom | Ju CKD TubInt | Protein expression |
|---|---|---|---|---|---|
| **Inhibitors** | **CKD cohort** | **CKD cohort** | **CKD cohort** | **CKD cohort** | **CKD cohort** |
| **Reversion-inducing cysteine-rich protein with Kazal motifs (RECK)** | Increase | Increase | Decrease | Not significant* | - |
| **Metalloproteinase inhibitor 1 (TIMP1)** | Increase | Not significant* | Increase | Increase | Increase [45] |
| **Metalloproteinase inhibitor 2 (TIMP2)** | Increase | Increase | Not significant* | Not significant* | - |
| **Metalloproteinase inhibitor 3 (TIMP3)** | Increase | Increase | Not significant* | Decrease | - |
| **Metalloproteinase inhibitor 4 (TIMP4)** | Decrease | Not significant* | Not significant* | Not significant* | - |
| **Alpha-2-macroglobulin (A2M)** | Not significant* | Not significant* | Increase | Increase | - |
| **Pregnancy zone protein (PZP)** | Decrease | Not significant* | Not significant* | Not significant* | - |
| **Testican-1 (SPOCK1)** | Not significant* | Not significant* | Decrease | Decrease | - |
| **Testican-3 (SPOCK3)** | Not significant* | Not significant* | Not significant* | Not significant* | - |
| **Serine protease inhibitor Kazal-type 6 (SPINK6)** | Increase | Not significant* | No data | No data | - |
| **Alpha-1-antitrypsin (SERPINA1)** | Increase | Not significant* | Not significant* | Not significant* | Increase [63] |
| **Antithrombin-III (SERPINC1)** | Decrease | Decrease | Decrease | Not significant* | inconclusive* |
| **Alpha-2-antiplasmin (SERPINF2)** | Decrease | Decrease | Decrease | Decrease | - |

Not significant*: $P > 0.05$, inconclusive*: some studies reported increased and some decreased protein expression levels in CKD.

https://doi.org/10.1371/journal.pone.0262667.t004

an anti-inflammatory mediator [41–43]. Given the abovementioned interactions of these proteases, the predicted decreased activities of all three *MMP2*, *9*, *14* in our study, is further enhanced, collectively suggesting for a more thorough investigation of these proteases in the CKD development and progression.

Four known enzymes namely *TIMP1* to *TIMP4* regulate either the activation and/or inhibition of MMPs. It is supported that the activity of the MMPs is regulated either intracellularly (transcriptional and posttranscriptional levels) and/or extracellularly (enzymatic activity). According to our results, we can conclude that the increased mRNA levels of TIMPs may lead to decreased activity of MMPs. Consequently, imbalances in the expression levels of MMPs and their inhibitors have been associated with essential cellular functions that lead to the development of CKD [43]. Even though the expression of MMPs and TIMPs in the kidneys has not been completely characterized, it has been reported that imbalanced levels of MMPs/TIMPs lead to dysregulated ECM formation/ breakdown and tissue remodelling [44]. Additionally, several cellular processes such as apoptosis, proliferation, inflammation, chemokine and cytokine production are regulated by MMPs/TIMPs imbalance, thus promoting the vascular, glomerular and tubular changes found in CKD [45]. Specifically, *MMP1*, *3*,*7* and *9* are mainly regulated by *TIMP1* while *TIMP2* is a major *MMP2* inhibitor [46]. Several studies have shown elevated levels of *TIMP1* in the urine and serum of CKD patients [47]. Furthermore, *TIMP1* may promote renal interstitial fibrosis [48]. In addition, both *TIMP1* and *TIMP2* are associated with glomerulosclerosis [5]. *TIMP3* is highly expressed in the kidney and inhibits mainly the activation of *MMP2*,*7* and *9* [49]. It has been shown that *TIMP3* may protect kidneys from damage [49, 50]. Although *TIMP4* expression in tissues is limited, studies support that it may inhibit *MMP14* leading to ECM deposition and inflammation [51]. Another reported inhibitor

of MMPs is the membrane-anchored protein RECK whose expression is increased by hyperglycemia [52]. An analysis showed that empagliflozin may reverse the renal suppression of RECK in human proximal tubular epithelial cells [52]. In summary, our data demonstrate that the TIMPs and RECK proteins have a distinct role in renal injury.

In addition to the MMPs, four proprotein convertases (*PCSK6*, *PCSK7*, *PCSK4* and *PCSK5*) were also predicted to be involved in the generation of the peptide data in our study. Two of these proprotein convertases have been previously associated with kidney disease: specifically, in the kidney, a high-salt diet induces the PCSK6-corin-ANP-AQP2/β-ENaC pathway suggesting the importance of *PCSK6* in renal failure [53], whereas *PCSK7* is linked to end-stage renal disease (ESRD) [54]. Notably, *PCSK6* is also considered a key regulator of smooth muscle cell function in vascular remodelling [55], a mechanism that is involved in CKD development. The role of *PCSK4* and *PCSK5* in CKD has not been investigated yet. However, the single-cell transcriptomic analysis revealed that *PCSK5* was differentially expressed in early human diabetic nephropathy [56]. Interestingly, *PSCK5*, predicted to be at increased activity in CKD based on our data and also supported by the mRNA Nephroseq expression pattern, has been reported as one of the enzymes involved in posttranslational processing and stability of fibroblast growth factor 23 (*Fgf23*), a protein which plays an important role in CKD development as it regulates phosphorus and vitamin D metabolism [57]. Nonetheless, knowledge of the inhibitors of the proprotein convertases is still very limited. Given our results, further investigation of *PCSK5* in CKD is warranted. Finally, *KLK4*, another serine protease with putative renoprotective properties [58], was predicted to have increased proteolytic activity in our study. Interestingly and in line with our prediction, decreased mRNA levels of the *KLK4* inhibitors *SERPINAC1* and *SERPINF2* have been observed in CKD, based on our analysis. Overall, 4 serine proteases including *SPINK6*, *SERPINA1*, *SERPINAC1* and *SERPINF2* inhibit the activity of *KLK4*. Several studies have reported the role of *SERPINF2* in the development of fibrosis [59, 60].

Among the strengths of our study are the large sample size and case-control matching, taking into consideration also the heart condition, suggesting high validity of our findings. Additionally, the same analytical platform (CE-MS) and protocols were used for the analysis of all urinary peptide datasets, leading to the generation of highly comparable data.

Limitations included that CKD and non-CKD individuals were acquired from different cohorts and studies. However, the large sample size, the case-control matching and the high significance of the findings reduce the risk of this bias. Additionally, the results of this study can only distinguish the effects of the two phases (CKD with eGFR $< 60$ ml/min/1.73 $m^2$ and non-CKD with eGFR $> 60$ ml/min/1.73 $m^2$) and cannot explain the molecular mechanisms of CKD development. We should however point that similar results were obtained when a different eGFR cut-off was applied (e.g. eGFR >70 versus eGFR <50; data not shown). Further, the weakness of our study is related to the fact that the predicted proteases are supported by the *in-silico* analysis and not by in vitro and/or in vivo experiments. It is expected that transcriptomics data and activity levels of the predicted proteases are not always in agreement. In addition, in the Nephroseq datasets, 'CKD' was not defined always in the same way, which may also explain the observed inconsistencies. However, our spherical approach, involving the cross-omics analysis, together with the in-depth literature mining generates a reliable shortlist of proteases and enhances the reliability of the observed agreements among the proteomics and multiple transcriptomics datasets opening up avenues for further investigation in future studies.

## Conclusions

In conclusion, our study revealed several interesting proteases including *MMP 7*, *14* and their interactions with *MMP2* and *MMP9* as well as *PCSK5* which may be involved in the generation

of CKD-associated urine peptides. These predicted proteases should be further investigated in the context of CKD development and progression and may be considered as putative therapeutic targets. With this study, we contribute to a better understanding of the proteolytic mechanisms involved in CKD and propose multiple proteases of possible significance in CKD pathogenesis.

## Supporting information

**S1 Table. List of the 303 Chronic kidney disease (CKD) differentially excreted peptides.**
(XLSX)

**S2 Table. Spearman's rank-order correlation analysis between the amplitude of the 303 CKD-associated peptides and eGFR of CKD patients.**
(XLSX)

**S3 Table. List of predicted proteases with calculated activity score.**
(XLSX)

## Author Contributions

**Data curation:** Eleni Petra.

**Formal analysis:** Eleni Petra.

**Methodology:** Eleni Petra.

**Software:** Eleni Petra.

**Supervision:** Antonia Vlahou, Joachim Jankowski.

**Validation:** Eleni Petra.

**Visualization:** Eleni Petra.

**Writing – original draft:** Eleni Petra.

**Writing – review & editing:** Eleni Petra, Justyna Siwy, Antonia Vlahou, Joachim Jankowski.

## References

1. Bikbov B, Purcell CA, Levey AS, Smith M, Abdoli A, Abebe M, et al. Global, regional, and national burden of chronic kidney disease, 1990–2017: a systematic analysis for the Global Burden of Disease Study 2017. The Lancet. 2020 Feb 29; 395(10225):709–33.
2. Stevens PE, Levin A. Evaluation and management of chronic kidney disease: Synopsis of the kidney disease: Improving global outcomes 2012 clinical practice guideline. Annals of Internal Medicine. 2013; 158(11):825–30. https://doi.org/10.7326/0003-4819-158-11-201306040-00007 PMID: 23732715
3. Gajjala PR, Sanati M, Jankowski J. Cellular and Molecular Mechanisms of Chronic Kidney Disease with Diabetes Mellitus and Cardiovascular Diseases as Its Comorbidities. Frontiers in immunology. 2015; 6:340. https://doi.org/10.3389/fimmu.2015.00340 PMID: 26217336
4. Webster AC, Nagler E v, Morton RL, Masson P. Chronic Kidney Disease. Lancet (London, England). 2017 Mar; 389(10075):1238–52.
5. Zakiyanov O, Kalousová M, Zima T, Tesař V. Matrix metalloproteinases in renal diseases: A critical appraisal. Kidney and Blood Pressure Research. 2019 Jun 1; 44(3):298–330. https://doi.org/10.1159/000499876 PMID: 31185475
6. Bond JS. Proteases: History, discovery, and roles in health and disease. The Journal of biological chemistry. 2019 Feb; 294(5):1643–51. https://doi.org/10.1074/jbc.TM118.004156 PMID: 30710012
7. Provenzano M, Andreucci M, Garofalo C, Faga T, Michael A, Ielapi N, et al. The association of matrix metalloproteinases with chronic kidney disease and peripheral vascular disease: A light at the end of the tunnel? Biomolecules. 2020 Jan 1; 10(1).

8. Peiskerová M, Kalousová M, Kratochvílová M, Dusilová-Sulková S, Uhrová J, Bandúr S, et al. Fibroblast growth factor 23 and matrix-metalloproteinases in patients with chronic kidney disease: Are they associated with cardiovascular disease? Kidney and Blood Pressure Research. 2009;32(4). https://doi.org/10.1159/000201792 PMID: 19212123
9. Cheng S, Pollock AS, Mahimkar R, Olson JL, Lovett DH, Cheng S, et al. Matrix metalloproteinase 2 and basement membrane integrity: a unifying mechanism for progressive renal injury. The FASEB Journal. 2006; 20(11):1898–900. https://doi.org/10.1096/fj.06-5898fje PMID: 16891619
10. Henger A, Kretzler M, Doran P, Bonrouhi M, Schmid H, Kiss E, et al. Gene expression fingerprints in human tubulointerstitial inflammation and fibrosis as prognostic markers of disease progression. Kidney International. 2004; 65(3).
11. Zhou D, Tian Y, Sun L, Zhou L, Xiao L, Tan RJ, et al. Matrix metalloproteinase-7 is a urinary biomarker and pathogenic mediator of kidney fibrosis. Journal of the American Society of Nephrology. 2017 Feb 1; 28(2):598–611. https://doi.org/10.1681/ASN.2016030354 PMID: 27624489
12. He W, Tan RJ, Li Y, Wang D, Nie J, Hou FF, et al. Matrix metalloproteinase-7 as a surrogate marker predicts renal Wnt/β-catenin activity in CKD. Journal of the American Society of Nephrology. 2012; 23 (2):294–304. https://doi.org/10.1681/ASN.2011050490 PMID: 22095947
13. Filip S, Pontillo C, Peter Schanstra J, Vlahou A, Mischak H, Klein J. Urinary proteomics and molecular determinants of chronic kidney disease: possible link to proteases. Expert review of proteomics. 2014 Oct; 11(5):535–48. https://doi.org/10.1586/14789450.2014.926224 PMID: 24957818
14. Rodríguez-Ortiz ME, Pontillo C, Rodríguez M, Zürbig P, Mischak H, Ortiz A. Novel Urinary Biomarkers For Improved Prediction Of Progressive Egfr Loss In Early Chronic Kidney Disease Stages And In High Risk Individuals Without Chronic Kidney Disease. Scientific reports. 2018 Oct; 8(1):15940. https://doi.org/10.1038/s41598-018-34386-8 PMID: 30374033
15. Pontillo C, Jacobs L, Staessen JA, Schanstra JP, Rossing P, Heerspink HJL, et al. A urinary proteome-based classifier for the early detection of decline in glomerular filtration. Nephrology, dialysis, transplantation: official publication of the European Dialysis and Transplant Association—European Renal Association. 2017 Sep; 32(9):1510–6. https://doi.org/10.1093/ndt/gfw239 PMID: 27387473
16. Good DM, Zürbig P, Argilés À, Bauer HW, Behrens G, Coon JJ, et al. Naturally occurring human urinary peptides for use in diagnosis of chronic kidney disease. Molecular and Cellular Proteomics. 2010; 9 (11):2424–37. https://doi.org/10.1074/mcp.M110.001917 PMID: 20616184
17. Krochmal M, Kontostathi G, Magalhães P, Makridakis M, Klein J, Husi H, et al. Urinary peptidomics analysis reveals proteases involved in diabetic nephropathy. Scientific reports. 2017 Nov; 7(1):15160. https://doi.org/10.1038/s41598-017-15359-9 PMID: 29123184
18. Markoska K, Pejchinovski M, Pontillo C, Zürbig P, Jacobs L, Smith A, et al. Urinary peptide biomarker panel associated with an improvement in estimated glomerular filtration rate in chronic kidney disease patients. Nephrology, dialysis, transplantation: official publication of the European Dialysis and Transplant Association—European Renal Association. 2018 May; 33(5):751–9. https://doi.org/10.1093/ndt/gfx263 PMID: 28992073
19. Schanstra JP, Zürbig P, Alkhalaf A, Argiles A, Bakker SJL, Beige J, et al. Diagnosis and prediction of CKD progression by assessment of urinary peptides. Journal of the American Society of Nephrology. 2015; 26(8):1999–2010. https://doi.org/10.1681/ASN.2014050423 PMID: 25589610
20. Argilés À, Siwy J, Duranton F, Gayrard N, Dakna M, Lundin U, et al. CKD273, a New Proteomics Classifier Assessing CKD and Its Prognosis. PLoS ONE. 2013; 8(5):e62837. https://doi.org/10.1371/journal.pone.0062837 PMID: 23690958
21. Gu YM, Thijs L, Liu YP, Zhang Z, Jacobs L, Koeck T, et al. The urinary proteome as correlate and predictor of renal function in a population study. Nephrology Dialysis Transplantation. 2014; 29(12):2260–8. https://doi.org/10.1093/ndt/gfu234 PMID: 24981581
22. Tofte N, Lindhardt M, Adamova K, Bakker SJL, Beige J, Beulens JWJ, et al. Early detection of diabetic kidney disease by urinary proteomics and subsequent intervention with spironolactone to delay progression (PRIORITY): a prospective observational study and embedded randomised placebo-controlled trial. The lancet Diabetes & endocrinology. 2020 Apr; 8(4):301–12. https://doi.org/10.1016/S2213-8587(20)30026-7 PMID: 32135136
23. Latosinska A, Siwy J, Mischak H, Frantzi M. Peptidomics and proteomics based on CE-MS as a robust tool in clinical application: The past, the present, and the future. Electrophoresis. 2019 Sep; 40(18–19):2294–308. https://doi.org/10.1002/elps.201900091 PMID: 31054149
24. Siwy J, Zürbig P, Argiles A, Beige J, Haubitz M, Jankowski J, et al. Noninvasive diagnosis of chronic kidney diseases using urinary proteome analysis. Nephrology, dialysis, transplantation: official publication of the European Dialysis and Transplant Association—European Renal Association. 2017 Dec; 32 (12):2079–89. https://doi.org/10.1093/ndt/gfw337 PMID: 27984204

**25.** He T, Mischak M, Clark AL, Campbell RT, Delles C, Díez J, et al. Urinary peptides in heart failure: a link to molecular pathophysiology. European Journal of Heart Failure. 2021 Nov 1; 23(11):1875–87. https://doi.org/10.1002/ejhf.2195 PMID: 33881206

**26.** Mischak H, Vlahou A, Ioannidis JPA. Technical aspects and inter-laboratory variability in native peptide profiling: the CE-MS experience. Clinical biochemistry. 2013 Apr; 46(6):432–43. https://doi.org/10.1016/j.clinbiochem.2012.09.025 PMID: 23041249

**27.** Jantos-Siwy J, Schiffer E, Brand K, Schumann G, Rossing K, Delles C, et al. Quantitative urinary proteome analysis for biomarker evaluation in chronic kidney disease. Journal of Proteome Research. 2009 Jan; 8(1):268–81. https://doi.org/10.1021/pr800401m PMID: 19012428

**28.** Klein J, Eales J, Zürbig P, Vlahou A, Mischak H, Stevens R. Proteasix: a tool for automated and large-scale prediction of proteases involved in naturally occurring peptide generation. Proteomics. 2013 Apr; 13(7):1077–82. https://doi.org/10.1002/pmic.201200493 PMID: 23348921

**29.** Zhou Y, Zhou B, Pache L, Chang M, Khodabakhshi AH, Tanaseichuk O, et al. Metascape provides a biologist-oriented resource for the analysis of systems-level datasets. Nature communications. 2019 Apr; 10(1):1523. https://doi.org/10.1038/s41467-019-09234-6 PMID: 30944313

**30.** Rawlings ND, Barrett AJ, Thomas PD, Huang X, Bateman A, Finn RD. The MEROPS database of proteolytic enzymes, their substrates and inhibitors in 2017 and a comparison with peptidases in the PANTHER database. Nucleic Acids Research. 2018; 46(D1). https://doi.org/10.1093/nar/gkx1134 PMID: 29145643

**31.** Nakagawa S, Nishihara K, Miyata H, Shinke H, Tomita E, Kajiwara M, et al. Molecular markers of tubulointerstitial fibrosis and tubular cell damage in patients with chronic kidney disease. PLoS ONE. 2015; 10(8). https://doi.org/10.1371/journal.pone.0136994 PMID: 26317775

**32.** Ju W, Nair V, Smith S, Zhu L, Shedden K, Song PXK, et al. Tissue transcriptome-driven identification of epidermal growth factor as a chronic kidney disease biomarker. Science Translational Medicine. 2015; 7(316). https://doi.org/10.1126/scitranslmed.aac7071 PMID: 26631632

**33.** Ju W, Greene CS, Eichinger F, Nair V, Hodgin JB, Bitzer M, et al. Defining cell-type specificity at the transcriptional level in human disease. Genome Research. 2013; 23(11):1862–73. https://doi.org/10.1101/gr.155697.113 PMID: 23950145

**34.** Ke B, Fan C, Yang L, Fang X. Matrix metalloproteinases-7 and kidney fibrosis. Vol. 8, Frontiers in Physiology. 2017.

**35.** Ślusarz A, Nichols LA, Grunz-Borgmann EA, Chen G, Akintola AD, Catania JM, et al. Overexpression of MMP-7 increases collagen 1A2 in the aging kidney. Physiological Reports. 2013; 1(5).

**36.** Zhou D, Tan RJ, Zhou L, Li Y, Liu Y. Kidney tubular β-catenin signaling controls interstitial fibroblast fate via epithelial-mesenchymal communication. Scientific Reports. 2013;3. https://doi.org/10.1038/srep01878 PMID: 23698793

**37.** Tan RJ, Li Y, Rush BM, Cerqueira DM, Zhou D, Fu H, et al. Tubular injury triggers podocyte dysfunction by β-catenin–driven release of MMP-7. JCI Insight. 2019; 4(24). https://doi.org/10.1172/jci.insight.122399 PMID: 31743113

**38.** Cheng Z, Limbu MH, Wang Z, Liu J, Liu L, Zhang X, et al. MMP-2 and 9 in chronic kidney disease. Vol. 18, International Journal of Molecular Sciences. 2017. https://doi.org/10.3390/ijms18040776 PMID: 28397744

**39.** Liu L, Li Y, Wang Z, Ding F, Cheng Z, Xu Q, et al. Rab7 empowers renal tubular epithelial cells with autophagy-mediated protection against albumin-induced injury. Experimental Cell Research. 2018; 370 (2):198–207. https://doi.org/10.1016/j.yexcr.2018.06.019 PMID: 29932881

**40.** Catania JM, Chen G, Parrish AR. Role of matrix metalloproteinases in renal pathophysiologies. Vol. 292, American Journal of Physiology—Renal Physiology. 2007. p. F905–11. https://doi.org/10.1152/ajprenal.00421.2006 PMID: 17190907

**41.** Tsai JP, Liou JH, Kao WT, Wang SC, Lian J da, Chang HR. Increased Expression of Intranuclear Matrix Metalloproteinase 9 in Atrophic Renal Tubules Is Associated with Renal Fibrosis. PLoS ONE. 2012; 7 (10). https://doi.org/10.1371/journal.pone.0048164 PMID: 23110201

**42.** Sanders JSF, Huitema MG, Hanemaaijer R, van Goor H, Kallenberg CGM, Stegeman CA. Urinary matrix metalloproteinases reflect renal damage in anti-neutrophil cytoplasm autoantibody-associated vasculitis. American Journal of Physiology—Renal Physiology. 2007; 293(6). https://doi.org/10.1152/ajprenal.00310.2007 PMID: 17898039

**43.** Hu Y, Ivashkiv LB. Costimulation of Chemokine Receptor Signaling by Matrix Metalloproteinase-9 Mediates Enhanced Migration of IFN-α Dendritic Cells. The Journal of Immunology. 2006; 176(10). https://doi.org/10.4049/jimmunol.176.10.6022 PMID: 16670311

44. Essick E, Sithu S, Dean W, D'Souza S. Pervanadate-induced shedding of the intercellular adhesion molecule (ICAM)-1 ectodomain is mediated by membrane type-1 matrix metalloproteinase (MT1-MMP). Molecular and Cellular Biochemistry. 2008; 314(1–2).

45. Hörstrup JH, Gehrmann M, Schneider B, Plöger A, Froese P, Schirop T, et al. Elevation of serum and urine levels of TIMP-1 and tenascin in patients with renal disease. Nephrology Dialysis Transplantation. 2002; 17(6). https://doi.org/10.1093/ndt/17.6.1005 PMID: 12032189

46. Nagase H, Visse R, Murphy G. Structure and function of matrix metalloproteinases and TIMPs. Vol. 69, Cardiovascular Research. 2006.

47. Lu Y, Liu S, Zhang S, Cai G, Jiang H, Su H, et al. Tissue inhibitor of metalloproteinase-1 promotes NIH3T3 fibroblast proliferation by activating p-Akt and cell cycle progression. Molecules and Cells. 2011; 31(3). https://doi.org/10.1007/s10059-011-0023-9 PMID: 21350939

48. Carome MA, Striker LJ, Peten EP, Moore J, Yang CW, Stetler-Stevenson WG, et al. Human glomeruli express TIMP-1 mRNA and TIMP-2 protein and mRNA. American Journal of Physiology—Renal Fluid and Electrolyte Physiology. 1993; 264(6 33–6). https://doi.org/10.1152/ajprenal.1993.264.6.F923 PMID: 8322893

49. Kassiri Z, Oudit GY, Kandalam V, Awad A, Wang X, Ziou X, et al. Loss of TIMP3 enhances interstitial nephritis and fibrosis. Journal of the American Society of Nephrology. 2009; 20(6). https://doi.org/10.1681/ASN.2008050492 PMID: 19406980

50. Wang Z, Famulski K, Lee J, Das SK, Wang X, Halloran P, et al. TIMP2 and TIMP3 have divergent roles in early renal tubulointerstitial injury. Kidney International. 2014; 85(1). https://doi.org/10.1038/ki.2013.225 PMID: 23760282

51. Takawale A, Fan D, Basu R, Shen M, Parajuli N, Wang W, et al. Myocardial recovery from ischemia-reperfusion is compromised in the absence of tissue inhibitor of metalloproteinase 4. Circulation: Heart Failure. 2014; 7(4). https://doi.org/10.1161/CIRCHEARTFAILURE.114.001113 PMID: 24842912

52. Das NA, Carpenter AJ, Belenchia A, Aroor AR, Noda M, Siebenlist U, et al. Empagliflozin reduces high glucose-induced oxidative stress and miR-21-dependent TRAF3IP2 induction and RECK suppression, and inhibits human renal proximal tubular epithelial cell migration and epithelial-to-mesenchymal transition. Cellular Signalling. 2020; 68.

53. Zhang J, Yin Y, Chen L, Chu C, Wang Y, Lv Y, et al. Short-Term High-Salt Diet Increases Corin Level to Regulate the Salt-Water Balance in Humans and Rodents. American Journal of Hypertension. 2018; 31 (2):253–60. https://doi.org/10.1093/ajh/hpx148 PMID: 28985241

54. Zeng X, Li C, Li Y, Yu H, Fu P, Hong HG, et al. A network-based variable selection approach for identification of modules and biomarker genes associated with end-stage kidney disease. Nephrology (Carlton, Vic). 2020 Oct; 25(10):775–84. https://doi.org/10.1111/nep.13655 PMID: 31464346

55. Rykaczewska U, Suur BE, Röhl S, Razuvaev A, Lengquist M, Sabater-Lleal M, et al. PCSK6 Is a Key Protease in the Control of Smooth Muscle Cell Function in Vascular Remodeling. Circulation research. 2020 Feb; 126(5):571–85. https://doi.org/10.1161/CIRCRESAHA.119.316063 PMID: 31893970

56. Wilson PC, Wu H, Kirita Y, Uchimura K, Ledru N, Rennke HG, et al. The single-cell transcriptomic landscape of early human diabetic nephropathy. Proceedings of the National Academy of Sciences [Internet]. 2019 [cited 2021 Sep 26]; 116(39):19619–25. Available from: https://www.pnas.org/content/116/39/19619 https://doi.org/10.1073/pnas.1908706116 PMID: 31506348

57. Liu S, Tang W, Fang J, Ren J, Li H, Xiao Z, et al. Novel regulators of Fgf23 expression and mineralization in Hyp bone. Molecular Endocrinology. 2009; 23(9):1505–18. https://doi.org/10.1210/me.2009-0085 PMID: 19556340

58. Krasoudaki E, Banos A, Stagakis E, Loupasakis K, Drakos E, Sinatkas V, et al. Micro-RNA analysis of renal biopsies in human lupus nephritis demonstrates up-regulated miR-422a driving reduction of kallikrein-related peptidase 4. Nephrology Dialysis Transplantation. 2016; 31(10):1676–86. https://doi.org/10.1093/ndt/gfv374 PMID: 26546590

59. Kanno Y, Kawashita E, Minamida M, Kaneiwa A, Okada K, Ueshima S, et al. α2-antiplasmin is associated with the progression of fibrosis. American Journal of Pathology. 2010; 176(1). https://doi.org/10.2353/ajpath.2010.090150 PMID: 20008146

60. Kanno Y, Kawashita E, Kokado A, Kuretake H, Ikeda K, Okada K, et al. α2AP mediated myofibroblast formation and the development of renal fibrosis in unilateral ureteral obstruction. Scientific Reports. 2014; 4. https://doi.org/10.1038/srep05967 PMID: 25095732

61. Feldreich T, Nowak C, Carlsson AC, Östgren CJ, Nyström FH, Sundström J, et al. The association between plasma proteomics and incident cardiovascular disease identifies MMP-12 as a promising cardiovascular risk marker in patients with chronic kidney disease. Atherosclerosis. 2020 Aug 1; 307:11–5. https://doi.org/10.1016/j.atherosclerosis.2020.06.013 PMID: 32702535

**62.** Enoksen IT, Svistounov D, Norvik J v, Stefansson VTN, Solbu MD, Eriksen BO, et al. Serum matrix metalloproteinase 7 and accelerated glomerular filtration rate decline in a general non-diabetic population. Nephrology Dialysis Transplantation. 2021 Aug 26;

**63.** Romanova Y, Laikov A, Markelova M, Khadiullina R, Makseev A, Hasanova M, et al. Proteomic analysis of human serum from patients with chronic kidney disease. Biomolecules. 2020; 10(2). https://doi.org/10.3390/biom10020257 PMID: 32046176
